# Association between the RETN -420C/G polymorphism and type 2 diabetes mellitus susceptibility: A meta-analysis of 23 studies

**DOI:** 10.3389/fendo.2022.1039919

**Published:** 2022-12-21

**Authors:** Fei Luo, Mingjie Shi, Junhao Guo, Yisen Cheng, Xusan Xu, Jieqing Zeng, Si Huang, Weijun Huang, Wenfeng Wei, Yajun Wang, Riling Chen, Guoda Ma

**Affiliations:** ^1^ Key Laboratory of Research in Maternal and Child Medicine and Birth Defects, Guangdong Medical University, Foshan, Guangdong, China; ^2^ Matenal and Child Research Institute, Shunde Women and Children’s Hospital (Maternity and Child Healthcare Hospital of Shunde Foshan), Guangdong Medical University, Foshan, China; ^3^ School of Public Health, Guangdong Medical University, Dongguan, China; ^4^ First College of Clinical Medicine, Guangdong Medical University, Zhanjiang, China; ^5^ Department of Internal Medicine, Shunde Women and Children’s Hospital (Maternity and Child Healthcare Hospital of Shunde Foshan), Guangdong Medical University, Foshan, China; ^6^ Department of Hematology-Oncology, Shunde Women and Children’s Hospital (Maternity and Child Healthcare Hospital of Shunde Foshan), Guangdong Medical University, Foshan, China

**Keywords:** type 2 diabetes mellitus, T2DM, resistin, RETN, polymorphism

## Abstract

**Background:**

The published findings on the link between the resistin (RETN) gene polymorphism and type 2 diabetes mellitus (T2DM) risk are still contradictory. Here, through a meta-analysis, we summarized a more precise evaluation of their connection by synthesizing existing research.

**Methods:**

PubMed, Google Scholar, and Web of Science were electronically searched, and all cited sources were manually searched. The heterogeneity of effects was tested and all statistical analyses were performed in Stata 12.0.

**Results:**

A total of 23 studies with 10,651 cases and 14,366 controls on RETN -420C/G polymorphism were included. The overall results showed that the association of RETN -420C/G polymorphism and T2DM susceptibility was not significant [for the allelic model: odds ratio (OR) = 0.98, 95% confidence interval (CI) = 0.87–1.10, p_heterogeneity_ <.001; *I* ^2^ = 84.6%; for the dominant model: OR = 0.96, 95% CI = 0.80–1.15, p_heterogeneity_ <.001; *I* ^2^ = 87.1%; and for the recessive model: OR = 0.96, 95% CI = 0.82–1.12, p_heterogeneity_ <.001; *I* ^2^ = 56.9%] but with high heterogeneity across studies (p <.0001). Meta-regression found that the median age of T2DM participants (using age 50 as the cutoff) could be a factor in the observed variation. The RETN -420C/G polymorphism seems to be linked to an increased risk of T2DM in younger individuals [for dominant: OR = 0.84 (95% CI, 0.72–0.98; p_heterogeneity_ <.001; *I* ^2^ = 80.9%)] and decreased risk in older people [for dominant: OR = 3.14 (95% CI, 2.35–4.19; p_heterogeneity_ = .98; *I* ^2^ = 0.0%)].

**Conclusions:**

Current results found no evidence that the RETN -420C/G variant was linked to T2DM susceptibility, but the patient’s age appears to be a potential factor that contributed to high heterogeneity across studies. Additional high-quality and well-designed investigations are required to confirm these results.

## 1 Introduction

Type 2 diabetes mellitus (T2DM) is an extremely prevalent polygenic disorder with a complex pathophysiology, a large number of complications, and a high mortality rate (1, 2). It is currently responsible for more than 90% of cases of diabetes worldwide and is projected to more than double by 2025 (3). T2DM is a chronic progressive disease characterized by impaired pancreatic β-cell function and insulin resistance in the liver, muscle, and adipose tissue (4). With the progressive identification of novel gene loci and disease-associated single nucleotide polymorphisms (SNPs) in T2DM, some of them have been shown to prevent or exacerbate disease progression (5).

Resistin (RETN), an adipocyte-derived peptide, belongs to a type of secretory protein rich in the amino acid, cysteine (6). Its serum levels have been shown to correlate with metabolic risk variables and insulin resistance, suggesting that resistin may play an important role in the pathogenesis of T2DM (7–9). The gene encoding resistin, RETN, is located on chromosome 19p13.3 14, where several SNPs have been identified, including -420C/G (rs1862513), 299G/A (rs3745367), and 62G/A (rs3745368). As one of the most frequently studied SNPs, -420C/G was found to increase the expression of resistin in blood and tissues by affecting the promoter activity, and therefore, was also found to have a role in controlling the onset of T2DM. The mRNA for this organism has a length of 476 base pairs (bp) with a 326-bp coding region that contains 108 amino acids. Recently, the importance of the RETN -420C/G locus in different worldwide populations was reported, either as a risk or a protective locus. For instance, Osawa et al. (9) originally suggested that RETN -420C/G may pose a substantial risk for T2DM susceptibility in the Japanese population, and this discovery was subsequently confirmed in external studies by Motawi et al. (10) and Nadeem et al. (11). In contrast, Ho et al. (12), Mohammadi-asl et al. (13), and Rathwa et al. (14) suggested that SNP in the -420C/G region has been linked to a reduced risk of T2DM. Moreover, most studies evaluating their association have found no association (15–19). These discrepancies may have resulted from insufficient sample size or inadequate coverage of the gene and its flanking regions. Here, we conducted a meta-analysis to systematically evaluate its relationship with T2DM susceptibility, which would help us better understand the role of the RETN -420C/G (rs1862513) polymorphism in T2DM risk.

## 2 Materials and methods

### 2.1 Search strategy

Following the Preferred Reporting Items for Systematic Reviews and Meta-Analyses (PRISMA) standards, we used a two-step procedure to identify relevant published papers. First, we searched PubMed, Google Scholar, and Web of Science to identify previous systematic reviews, and scrutinized the reference lists from relevant reviews. Second, we searched PubMed and other databases for full-text articles describing trials to retrieve all articles that have investigated the relationship of RETN -420C/G (rs1862513) for T2DM susceptibility by using the following terms: (a) “resistin” or “RETN” and “polymorphism” or “genotype” or “-420C/G” or “rs1862513” and (b) “type 2 diabetes mellitus” or “type 2 diabetes” or “diabetes mellitus” or “diabetic patients” or “T2DM”. The bibliographic references of the retrieved articles were also systematically reviewed for additional studies on RETN -420C/G. Meanwhile, the records were screened by assessing titles and abstracts, and thereafter, retrieved in full text and judged according to eligibility criteria.

### 2.2 Study selection and exclusion criteria

All eligible studies in this meta-analysis were required to satisfy the following criteria: case-control studies that (1) examined the connection between RETN -420C/G and T2DM (2), had data sufficient for quantitative analysis [such as the number of genotypes or the calculated odds ratios (ORs) with 95% confidence intervals (CIs)], and (3) were published in English. Studies that were duplicates, had a lack of clarity in defining the case or control groups, or were written in a language other than English or on subjects other than humans were disqualified.

### 2.3 Statistical analysis

A meta-analysis was used to calculate the strength of the association between the -420C/G SNP of the RETN gene and T2DM risk. We calculated pooled ORs with 95% CIs and assessed heterogeneity using the p-value and *I* ^2^ statistic. A random-effects model (Der Simonian and Laird) was used for the meta-analysis if there was heterogeneity caused by a non-threshold effect (p <.10 or *I* ^2^ > 50%), otherwise a fixed-effects model (Mantel-Haenszel) would be applied (p >.10 or *I* ^2^ < 50%). An *I* ^2^ > 50% indicated moderate to high heterogeneity. Therefore, we performed meta-regression and subgroup analyses to detect the potential sources of heterogeneity. Further, publication biases were detected using the Begg’s rank correlation test and Egger’s linear regression test; p <.05 indicated significance. All statistical analyses were performed using the Stata (version 12.0) and RevMan (version 5.3.0) software packages.

## 3 Results

### 3.1 Data selection and characteristics of the studies

**Figure 1:**
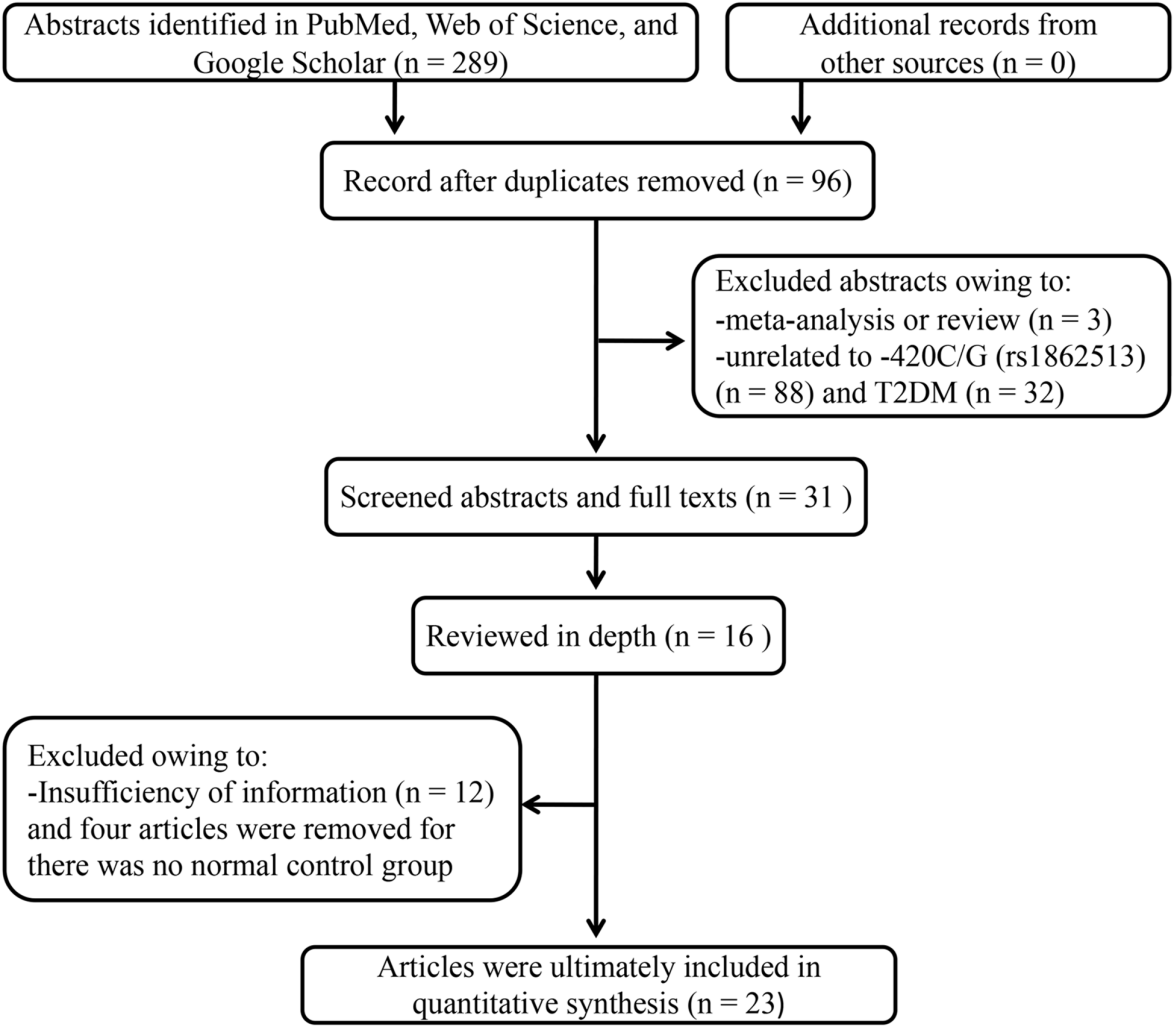
Flow diagram of the Preferred Reporting Items for Systematic Reviews and Meta-Analyses (PRISMA) study selection.

The literature search resulted in 93 studies meeting the keyword criteria (**Figure 1**). Removal of duplicates, commentary, letters, and basic research articles yielded 23 full-text articles with 10,651 cases and 14,366 controls for inclusion. The median sample sizes were 410 (42–2610) and 553 (49–3189) for patients and healthy controls, respectively. Among them, 12 originated in Asia (9, 11, 12, 14, 17, 19–25), five in the Americas (16, 18, 26–28), five in Europe (13, 15, 29–31), and one in Africa (10). Fifteen studies used non-diabetic controls (9, 14–18, 20–24, 26, 27, 30, 31), while the remaining eight relied on healthy-based controls (10–13, 19, 25, 28, 29). The main characteristics of the studies are summarized in **Table 1**.

**Table 1:** Characteristics of the investigated studies on the association between the resistin (RETN) -420C/G gene polymorphism and type 2 diabetes mellitus (T2DM) susceptibility.

Author(year, country)	Genotype method	Sample size	Age(case/control, year)	Gender(case/control M%)	Source of controls	Control			Case		
						CC	CG	GG	CC	CG	GG
Engert (2002, Canada)	PCR	179/180	52 (mean)	NA	NB	103	69	8	90	78	11
Engert (2002, Scandinavia)	PCR	452/433	61 (mean)	NA	NB	236	156	41	238	170	44
Ma (2002, Canada)	PCR	312/303	61 ± 6/62 ± 14	54.2/51.8	NB	140	134	24	148	128	24
Cho (2004, Korea)	PCR	411/173	59 ± 10/65 ± 4	47.9/34.7	NB	89	63	21	194	163	54
Osawa (2004, Japan)	PCR	546/564	59 ± 12/61 ± 9	57.9/50.7	NB	247	269	48	216	254	76
Kunnari (2004, Finland)	PCR	258/494	58 ± 16/51 ± 6	48.8/51.9	Healthy	266	197	29	151	85	18
Bouchard (2004, Canada)	NA	42/683	NA	41.8^a^	NB	337	256	90	22	17	3
Ochi (2007, Japan)	PCR	2,610/2,502	NA	NA	NB	1,080	1,123	299	1,169	1,144	297
Cauchi (2008, France)	PCR	1,244/3,189	60 ± 11/47 ± 12	64.7/42.6	NB	1,499	1,249	291	590	533	107
Tsukahara (2009, Japan)	PCR	349/286	57 ± 32/55 ± 25	55.3/59.8	NB	116	130	40	155	147	47
Chi (2009, China)	PCR-RFLP, DHPLC	318/370	58 ± 11/59 ± 9	56.3/60.5	Healthy	54	178	137	48	160	111
Emamgholipour (2009, Iran)	PCR	47/66	59 ± 9/59 ± 9	69.9^a^	NB	16	41	9	23	16	8
Lau (2010, Malaysia)	RT-PCR	427/280	50 ± 10^a^	100.0/100.0	NB	70	104	34	147	220	60
Chen (2010, USA)	PCR	529/529	61 ± 7/61 ± 7	32.1/32.1	NB	245	223	50	246	225	49
Hishida (2013, Japan)	mPCR-based invader	161/2,490	52 ± 17^a^	66.5/50.2	Healthy	1,062	1,087	341	76	74	11
Motawi (2014, Egypt)	RCR-RFLP	90/60	50 ± 8/47 ± 8	38.0/42.0	Healthy	31	22	7	21	35	34
Tellez (2016, Mexico)	PCR	45/49	54 ± 7/35 ± 10	26.7/22.4	Healthy	37	7	2	37	7	1
Ho (2017, China)	PCR	305/244	58 ± 11/52 ± 13	52.1/63.9	Healthy	70	127	47	124	139	42
Nadeem (2018, Pakistan)	RCR-RFLP	539/250	43 ± 5/43 ± 4	51.0/49.0	Healthy	114	32	4	202	322	15
Mohammadi-asl (2019, Iran)	RCR-RFLP	200/200	61 ± 6/62 ± 5	24.0/26.5	Healthy	42	135	33	102	66	32
Rathwa (2019, India)	RCR-RFLP	469/382	56 ± 10/49 ± 10	53.9/49.5	NB	63	188	131	172	204	93
Altawallbeh (2021, Jordan)	PCR	242/125	54 ± 9/52 ± 11	47.5/51.2	NB	35	73	17	95	161	26
Suriyaprom (2008, Thailand)	RCR-RFLP	95/105	57 ± 3/55 ± 3	NA	NB	51	54	35	60
Conneely (2004, Finland)	MassEXTEND	781/409	64 ± 8/66 ± 7	55.7/41.1	NB	–	–	–	–	–	–

PCR-RFLP, polymerase chain reaction-restriction fragment length polymorphism; DHPLC, denaturing high-performance liquid chromatography; NB, non-diabetic; T2DM, type 2 diabetes mellitus; and NA, not available. ^a^This represents the age of all subjects or the percentage of male patients.

### 3.2 Quantitative synthesis

**Figure 2:**
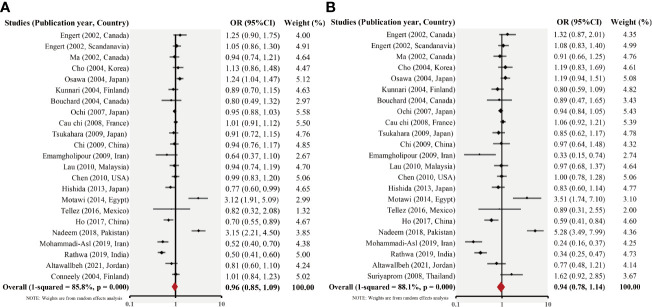
Forest plot of the allelic- (G vs. C) **(A)** and dominant (CG + GG vs. CC) **(B)** models for the 23 studies.

In general, no significant connection between the RETN -420C/G gene polymorphism and T2DM susceptibility was observed when data from all 23 studies were pooled. The allelic model of inheritance proposes that the frequency values of the G allele were 0.506 and 0.517 for patients with T2DM and for those without, respectively. The pooled OR for RETN G allelic frequency was 0.98 (95% CI, 0.87–1.10, p_heterogeneity_ <.001; *I* ^2^ = 84.6%). Similarly, the dominant model of inheritance exhibited CG + GG vs. CC values of 1.295 in the T2DM group and 1.269 in the control group. The pooled OR for the CG + GG vs. CC value was 0.96 (95% CI, 0.80–1.15; p_heterogeneity_ <.001; *I* ^2^ = 87.1%). Similarly, the results of other models are not significantly different [recessive model (CC vs. CG + GG): OR= 0.96 (95% CI, 0.82–1.12), p_heterogeneity_ <.001; *I* ^2^ = 56.9%; overdominant model (GG + CC *vs*. CG): OR = 1.13, 95% CI = 0.88–1.19, p_heterogeneity_ <.001; *I* ^2^ = 81.4%; homozygous model (GG vs. CC): OR = 0.91, 95% CI = 0.73–1.13, p_heterogeneity_ <.001; *I* ^2^ = 75.0%; and heterozygous model (CG *vs*. CC): OR = 0.94, 95% CI = 0.78–1.13, p_heterogeneity_ <.001; *I* ^2^ = 86.0%). The primary meta-analysis findings are displayed in **Figure 2** and **Table 2**, respectively.

**Table 2:** The meta-regression results based on the dominant model involving 23 studies.

Category	Coefficient	Standard error	T value	p Value	95% CI
Country	0.0260395	0.1201550	0.22	0.831	-0.2300648 to 0.2821438
Publication year	0.0390553	0.4539436	0.09	0.933	-0.9285025 to 1.0066130
Genotype method	0.0003421	0.3199269	0.00	0.999	-0.6815659 to 0.6822502
The ratio of T2DM and control group size	-0.1727046	0.3704861	-0.47	0.648	-0.9623771 to 0.6169680
Mean age (T2DM)	0.9442923	0.4257894	2.22	0.042^*^	0.0367437 to 1.8518410
Mean age (Control)	-0.7397364	0.4178218	-1.77	0.097	-1.6303020 to 0.1508296
Source of controls	-0.0545133	0.4104800	-0.13	0.896	-0.9294307 to 1.8091260
Summation	0.0210535	0.8586547	-0.02	0.981	-1.8512330 to 1.8091260

*P value < .05.

Moreover, we discovered substantial heterogeneity among the six models. To detect the potential factors that contributed to heterogeneity, we used meta-regression to investigate the possible causes of variation within six genetic models. Factors that could have affected the results were also included, such as the country or location of the study, publication year, genotype method, the ratio of participants in the T2DM group versus the control group, the mean age of the T2DM and control groups, and the source of control. Only the T2DM group’s average age, however, showed the possibility of explaining heterogeneity (p = .04) in the allelic and dominant models (**Table 2** and **Supplementary Table S1**).

**Figure 3:**
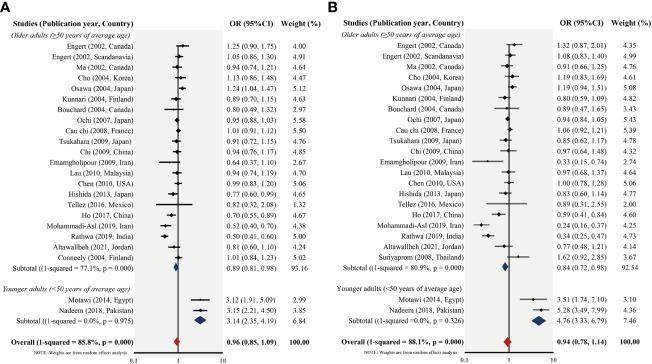
Forest plot of the allelic- (G vs. C) **(A)** and dominant (CG + GG vs. CC) **(B)** models stratified by the type 2 diabetes mellitus (T2DM) group mean age for the resistin (RETN) -420C/G polymorphism and T2DM susceptibility.

Studies using the T2DM group’s mean age of 50 as the cutoff were then divided into subsections 1 and 2, respectively. In subsection 1 of 24 studies, the pooled OR for the G *vs*. C value was 0.89 (95% CI, 0.83–0.98; p_heterogeneity_ <.001; *I* ^2^ = 77.1%) and for the CG + GG *vs*. CC value, it was 0.84 (95% CI, 0.72–0.98; p_heterogeneity_ <.001; *I* ^2^ = 80.9%), respectively. In subsection 2 of two studies, the pooled OR for the G *vs*. C value was 3.14 (95% CI, 2.35–4.19; p_heterogeneity_ = .98; *I* ^2^ = 0.0%) and for the CG + GG *vs*. CC value, it was 4.76 (95% CI, 3.33–6.79; p_heterogeneity_ = .33; *I* ^2^ = 0.0%), respectively. Overall, the results of subsection analysis showed that the RETN -420C/G polymorphism seemed to be linked to an increased risk of T2DM in younger persons and a lower risk in elderly patients (**Figure 3** and **Table 3**). Yet studies within subsection 1 had substantial heterogeneity across studies (p <.0001), and additional high-quality and well-designed investigations are required to corroborate these results.

**Table 3:** Meta-analyses of the resistin (RETN) -420C/G gene polymorphism and the risk of type 2 diabetes mellitus (T2DM) in subgroups stratified by the patients’ mean age.

Category	N	Allelic^a^		Recessive^b^		Dominant^c^		Overdominant^d^		Additive (Homozygous)^e^		Additive (Heterozygous)^f^	
		OR (95%)	I^2^ (%)	OR (95%)	I^2^ (%)	OR (95%)	I^2^ (%)	OR (95%)	I^2^ (%)	OR (95%)	I^2^ (%)	OR (95%)	I^2^ (%)
**Overall**	23^a,c^/ 22^b,c,d,e,f^	0.96 (0.85–1.09)	85.8*	0.93 (0.79–1.10)	60.2*	0.94 (0.78–1.14)	88.1*	1.13 (0.87–1.21)	83.6*	0.87 (0.69–1.09)	77.0*	0.92 (0.75–1.12)	79.7*
Subsection by T2DM group age													
Subsection 1: age >50	21^a,c^/ 20^b,c,d,e,f^	0.89 (0.81, 0.98)*	77.1*	0.89 (0.77, 1.04)	52.6*	0.84 (0.72, 0.98)*	80.9*	1.11 (0.96, 1.25)	69.5*	0.79 (0.64–0.98)*	73.2*	0.82 (0.69–0.96)*	87.7*
Subsection 2: age ≤50	2^a,b,c,d,e,f^	3.14 (2.35, 4.19)*	0.0	2.28 (0.53, 9.74)	75.7*	4.76 (3.33, 6.79)*	0.0	0.40 (0.08, 1.92)	93.6	4.02 (1.22, 13.29)*	61.0	3.88 (1.64, 9.14)*	74.1*

N, the number of included studies, including (a) allelic, (b) recessive, (c) dominant, (d) overdominant, (e) homozygous, and (f) heterozygous; T2DM, type 2 diabetes mellitus. *This means that there is significant heterogeneity between studies (p < .05).

### 3.3 Publication bias

**Figure 4:**
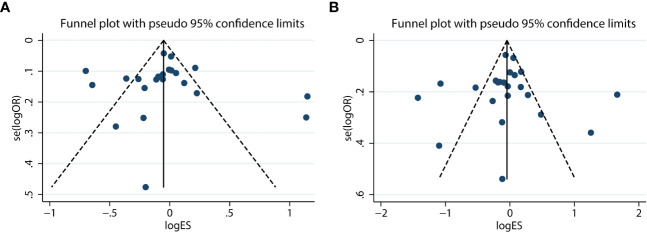
Funnel plot of the allelic (G vs. C) **(A)** and dominant (CG + GG vs. CC) **(B)** models for the resistin (RETN) -420C/G polymorphism and type 2 diabetes mellitus (T2DM) susceptibility.

Additionally, Begg’s and Egger’s tests were applied to evaluate their publication bias. There was minimal to no evidence of publication bias in either the allelic or dominant models, as indicated by the symmetrical funnel plots (**Figure 4**). Similarly, the results of other models on funnel plots were not significantly different (data not shown). There was also no evidence of publication bias provided by Begg’s and Egger’s tests (allelic model, p_Begg’s_ = .85, p_Egger’s_ = .49; dominant model, p_Begg’s_ = .86, p_Egger’s_ = .79; recessive model, p_Begg’s_ = .61, p_Egger’s_ = .87; overdominant model, p_Begg’s_ = .82, p_Egger’s_ = .98; homozygous model, p_Begg’s_ = .97, p_Egger’s_ = .74; and heterozygous model, p_Begg’s_ = .68, p_Egger’s_ = .70).

## 4 Discussion

Mounting evidence has shown that genetic variants may be associated with the risk of multiple human diseases. The relationship between the variant -420C/G (rs1862513) in the RETN locus and T2DM remains partially controversial. For instance, Osawa et al. (9) found that individuals who carried more than one copy of the haplotype G versus none had a significant association with T2DM susceptibility, while it was not consistently observed in the other two panels. In contrast, Ho et al. (12), Mohammadi-asl et al. (13), and Rathwa et al. (14) showed that individuals carrying the RETN -420C/G G allele or GG genotype had a significantly diminished risk of T2DM since their OR values were below 1.0. Additionally, some studies suggested that the -420C/G (rs1862513) polymorphism was not related to the T2DM risk. These inconsistent results forced us to elucidate the link between the rs1862513 polymorphism and T2DM risk by meta-analysis. Our present meta-analysis of 23 case-control studies (10,651 cases and 14,366 controls) provided the most comprehensive analysis of the relationship between the RETN -420C/G polymorphism and the risk of T2DM. The findings suggested that RETN -420C/G (rs1862513) was not related to overall T2DM susceptibility but with high heterogeneity across studies (p <.0001).

Between-study heterogeneity, which plagues the present results and many other meta-analyses of genetic association research, casts doubt on the trustworthiness of the findings. Common reasons for heterogeneity may be attributed to the diversity in design, participant characteristics, sample sizes, genotyping method, among others, and investigating the probable causes of heterogeneity is the essential component of meta-analysis. In our study, we found significant heterogeneity in all the six models (allelic: *I* ^2^ = 84.6%, p_heterogeneity_ <.001; dominant: *I* ^2^ = 87.1%, p_heterogeneity_ <.001; recessive: *I* ^2^ = 56.9%, p_heterogeneity_ <.001; overdominant: *I* ^2^ = 81.4%, p_heterogeneity_ <.001; homozygous: *I* ^2^ = 75.0%, p_heterogeneity_ <.001; and heterozygous: *I* ^2^ = 86.0%, p_heterogeneity_ <.001). To explore the sources of heterogeneity, we first used meta-regression to investigate the possible drivers of variation within the dominant genetic model. Confounding factors, such as study country, publication year, genotype method, the ratio of participants in the T2DM group versus the control group, and the mean age of the T2DM and control groups were included. Only the T2DM group’s average age, however, showed promise in explaining heterogeneity, implying that the age composition of T2DM subjects contributed to the heterogeneity across studies. The RETN -420C/G polymorphism might be mediated by age, and its variant seems to be linked to an increased risk of T2DM in younger people but a lower risk in older patients. Even after accounting for the age structure of the T2DM cohort, there was still unexplained heterogeneity in the younger T2DM population. There needs to be more high-quality, well-designed studies done to verify these findings.

In addition, we sought out one meta-analysis and systematic review of -420C/G and discovered that its results considerably differed from ours due to discrepancies in the number and methodology of the included studies. Wen et al. (32) pooled data from 12 studies published before 2013, including analyses stratified by race and control source, and found no connection between the RETN -420C/G polymorphism and the development of T2DM. In this study, we identified that the age of individuals with T2DM was a significant factor contributing to heterogeneity by increasing the number of studies and analyzing confounding factors. Nevertheless, some inevitable deficiencies of this meta-analysis should be recognized for proper interpretation of the results. First, RETN -420C/G (rs1862513) was revealed to have peptidase activity; its link with T2DM may also be influenced by environmental factors such as dietary food preferences, as numerous studies have established the crucial role of homocysteine in biochemical processes in T2DM progression (33, 34). Given the scarcity of relevant clinical data, it is currently impossible to say whether this polymorphism influences the severity of T2DM. Furthermore, as a form of retrospective research, meta-analysis might be affected by biases in recall and selection. It is also important to highlight that the results of the present meta-analysis may be influenced by the heterogeneity between studies. More so, there were nine studies that had sample sizes of less than 500, and a meta-analysis of 13 research studies looking at the effects of small trials indicated that they, on average, had better results. Consequently, results from small trials should be interpreted with caution, especially when they lack high methodological quality (35). Given these caveats, our results should be interpreted with caution until larger, better-designed trials are conducted.

In conclusion, studies examining the correlation between RETN -420C/G (rs1862513) and T2DM have been popular for decades, but the results have varied widely. Current evidence showed that the association of RETN -420C/G polymorphism and T2DM susceptibility was not significant but had high heterogeneity across studies. By examining potential confounders, the RETN-420C/G polymorphism may be age-mediated, and its variants appear to be associated with an increased risk of T2DM in younger people but with an opposite effect in older individuals. This finding should be regarded with caution, as there are not enough available studies to draw firm conclusions, and the present data are highly heterogeneous. More high-quality, well-designed studies are needed to shed light on the prevalence of -420C/G in T2DM patients of varying ages, as well as the effect of this distribution on the susceptibility of T2DM patients with and without comorbidities. Only then will we be able to fully comprehend how -420C/G polymorphism functions within the pathogenesis of T2DM.

## Data availability statement

The original contributions presented in the study are included in the article/**Supplementary Material**. Further inquiries can be directed to the corresponding authors.

## Author contributions

FL and MS conceived and drafted this meta-analysis. JG, YC, XX and JZ conducted the literature search. SH, WH, WW and YW performed the data extraction, quality assessment, and statistical analysis. RC and GM critically revised the manuscript. All authors contributed to the article and approved the submitted version.
