# Supplementary material for: Association between the RETN -420C/G polymorphism and type 2 diabetes mellitus susceptibility: A meta-analysis of 23 studies
Source: Front Endocrinol (Lausanne). 2022 Dec 21;13:1039919. doi: 10.3389/fendo.2022.1039919 (PMC9810749; doi:10.3389/fendo.2022.1039919)
Supplement: Supplementary file 1 [file Table_1.docx]

**Table S1.** The meta-regression results are based on other models involving 23 studies.

| **Category** | | **Coefficient** | **Standard error** | **T value** | **P value** | **95% CI** |
| --- | --- | --- | --- | --- | --- | --- |
| **Allele**  **(G vs C)** | Country | 0.0705082 | 0.0742708 | 0.95 | 0.357 | -0.0877964 to 0.2288127 |
|  | Publication year | 0.0514405 | 0.2816051 | 0.18 | 0.858 | -0.5487866 to 0.6516676 |
|  | Genotype method | -0.0371263 | 0.1971067 | -0.19 | 0.853 | -0.4572494 to 0.3829967 |
|  | Ratio of T2DM and control group size | -0.0599689 | 0.2296605 | -0.26 | 0.798 | -0.5494786 to 0.4295408 |
|  | Mean age (T2DM) | 0.6831230 | 0.2681779 | 2.55 | 0.022 | 0.1115153 to 1.2547310 |
|  | Mean age (Control) | -0.5558250 | 0.2625753 | -2.12 | 0.051 | -1.1154910 to 0.0038411 |
|  | Source of controls | -0.0189134 | 0.2527749 | -0.07 | 0.941 | -0.5576904 to 0.5198637 |
|  | Summation | -0.2069386 | 0.5295675 | -0.39 | 0.701 | -1.3356850 to 0.9218079 |
| **Recessive**  **(CC vs CG + GG)** | Country | 0.1464960 | 0.7285480 | 2.01 | 0.064 | -0.0097620 to 0.3027539 |
|  | Publication year | -0.0111054 | 0.2941557 | -0.04 | 0.970 | -0.6420068 to 0.6197959 |
|  | Genotype method | -0.1569805 | 0.2101622 | -0.75 | 0.467 | -0.6077337 to 0.2937727 |
|  | Ratio of T2DM and control group size | -0.1666977 | 0.2571522 | -0.65 | 0.527 | -0.7182342 to 0.3848389 |
|  | Mean age (T2DM) | 0.5862010 | 0.3062645 | 1.91 | 0.076 | -0.0706710 to 1.2430730 |
|  | Mean age (Control) | -0.5334210 | 0.2913642 | -1.83 | 0.088 | -1.1583350 to 0.0914931 |
|  | Source of controls | -0.0844565 | 0.2908584 | -0.29 | 0.776 | -0.7082857 to 0.5393726 |
|  | Summation | 0.2211906 | 0.5751051 | 0.38 | 0.706 | -1.0122870 to 1.4546680 |
| **over-dominant ((CC+GG) VS CG)** | Country | 0.1086046 | 0.1109327 | 0.98 | 0.345 | -0.1310551 to 0.3482602 |
|  | Publication year | -0.2176384 | 0.4171973 | -0.52 | 0.611 | -1.1189380 to 0.6836615 |
|  | Genotype method | -0.0723905 | 0.3024627 | -0.24 | 0.815 | -0.7258214 to 0.5810405 |
|  | Ratio of T2DM and control group size | 0.0238658 | 0.3878761 | 0.62 | 0.549 | 0.5992979 to 1.0766130 |
|  | Mean age (T2DM) | -0.6380174 | 0.3835598 | -1.66 | 0.12 | -1.4666480 to 0.1906133 |
|  | Mean age (Control) | 0.4947005 | 0.3913693 | 1.26 | 0.228 | -0.3508014 to 1.3402020 |
|  | Source of controls | 0.2017364 | 0.4081164 | 0.49 | 0.629 | -0.6799455 to 1.0834180 |
|  | Summation | -0.3768078 | 0.9505845 | -0.40 | 0.698 | -2.4304210 to 1.6768050 |
| **Additive (Homozygous, GG VS CC)** | Country | 0.1652504 | 0.1139171 | 1.45 | 0.169 | -0.0790774 to 0.4095782 |
|  | Publication year | -0.2207660 | 0.4490105 | -0.49 | 0.631 | -1.1837980 to 0.7422657 |
|  | Genotype method | -0.2239410 | 0.3324008 | -0.67 | 0.511 | -0.9368698 to 0.4889878 |
|  | Ratio of T2DM and control group size | -0.0851319 | 0.3863354 | -0.22 | 0.829 | -0.9137390 to 7434752 |
|  | Mean age (T2DM) | 0.8655051 | 0.4499553 | 1.92 | 0.075 | -0.0995531 to 1.8305630 |
|  | Mean age (Control) | -0.7151867 | 0.4431124 | -1.61 | 0.129 | -1.6655680 to 0.2351949 |
|  | Source of controls | 0.0110191 | 0.4370029 | 0.03 | 0.98 | -0.9262589 to 0.9482971 |
|  | Summation | 0.0696385 | 0.8797368 | 0.08 | 0.938 | -1.8172090 to 1.9564860 |
| **Additive (Heterozygote, GC VS CC)** | Country | -0.0230023 | 0.1365261 | -0.17 | 0.869 | -0.3179491 to 0.2719444 |
|  | Publication year | 0.0019897 | 0.5109273 | 0.00 | 0.997 | -1.1018020 to 1.1057810 |
|  | Genotype method | -0.0526186 | 0.3708959 | -0.14 | 0.889 | -0.8538904 to 0.7486533 |
|  | Ratio of T2DM and control group size | -0.0932876 | 0.4723324 | -0.20 | 0.846 | -1.1137000 to 0.9271245 |
|  | Mean age (T2DM) | 0.8403007 | 0.4670844 | 1.80 | 0.095 | -0.1687737 to 1.8493750 |
|  | Mean age (Control) | -0.5856319 | 0.4760874 | -1.23 | 0.24 | -1.6141560 to 0.4428923 |
|  | Source of controls | 0.0432457 | 0.4980559 | 0.09 | 0.932 | -1.0327390 to 1.1192300 |
|  | Summation | -0.1445762 | 1.1596540 | -0.12 | 0.903 | -2.6498570 to 2.3607050 |
